# Editorial: Expanding insights into structure, function, and disorder of genome by the power of artificial intelligence in bioinformatics

**DOI:** 10.3389/fgene.2025.1649410

**Published:** 2025-07-28

**Authors:** Hongqiang Lyu, Yao Li, Sijia Wu, Laiyi Fu, Xiaoping Liang, Erhu Liu

**Affiliations:** ^1^ School of Automation Science and Engineering, Faculty of Electronic and Information Engineering, Xi’an Jiaotong University, Xi’an, Shaanxi, China; ^2^ School of Life Science and Technology, Xidian University, Xi’an, Shaanxi, China; ^3^ The Solomon H. Snyder Department of Neuroscience, Johns Hopkins University School of Medicine, Baltimore, MD, United States; ^4^ School of Information and Control Engineering, Xi’an University of Architecture and Technology, Xi’an, Shaanxi, China

**Keywords:** artificial intelligence, machine learning, bioinformatic analysis, topologically associated domains, biomarker discovery, co-expression analysis, DNA barcoding

High-throughput sequencing technology allows to sequence genome expeditiously on an unprecedented scale by leveraging the potential of managing millions of fragments simultaneously. Besides, the boom of microfluidics and combinatorial index strategies give rise to single-cell technology, enabling high-throughput analysis of individual cells. These technologies produce a large amount of genomic and transcriptomic data for tens of thousands of individual cells in each experiment. The data provides a way to investigate cell-cell variations that are often obscured while studying at bulk level, and gives a chance for getting deeper insights into the structure, function, and disorder of genome. However, the problem that comes with it is how to obtain the biologically meaningful knowledge underlying the large-scale data, which poses challenges to traditional statistical methods, since the data at single-cell level has a large sample size, seems ultra-sparse coupled with noise, artifacts, and dropout events from experiments, and exhibits cell heterogeneity caused by cell cycle and transcription status. Fortunately, the advancements of machine learning (ML), especially artificial intelligence methods, contribute to bioinformatic analysis of large-scale genomic and transcriptomic data, especially at single-cell level: 1) Complex patterns and nonlinear relationships can be discovered via the training procedure on a large number of samples, and it happens that the sample size at single-cell level is huge. 2) Hierarchical feature representations can be automatically learned, and the feature interactions are considered implicitly, which help to handle noise, outliers, and redundancies in data. 3) Adaptive models can be created without relying on prior knowledge, such as data distribution, the model structure is gradually adjusted during the optimization of parameters, especially in the case of end-to-end deep learning (DL) network. In addition, the recent rises of graph neural network and multimodal DL have also been promoting the computational analysis of ultra-sparse and multi-omics data. It can be seen that ML, especially DL methods, are promising, particularly with the advancement of single-cell experimental techniques and the accumulation of large amounts of omics data. In view of this, our Research Topic tries to collect some new advances in ML, especially DL methods, which may contribute to expand insights into structure, function, and disorder of genome by the power of artificial intelligence in bioinformatics.

The advantages of artificial intelligence have been highlighted while entering the bioinformatic analysis of spatial organizations of genome at single-cell level, especially Topologically Associated Domains (TADs). The TADs discovered on bulk Hi-C data are regarded as fundamental building blocks of three-dimensional genome. Structure affects function, TADs effectively participate in the regulatory programs of gene expression, and have received continuous attention while stepping into the era of single-cell omics. The bioinformatic analysis of TADs on scHi-C data is expected to tell us more compared with that on bulk Hi-C data. Lyu et al. conducted a survey of artificial intelligence involved in bioinformatic tools and applications for TADs on single-cell Hi-C data, including imputation of scHi-C data, identification of TAD boundaries and hierarchy, and differential analysis of TAD structures. The categories, characteristics, and evolutions of the latest available methods were summarized, and the artificial intelligence strategies involved in these Research Topic were particularly dissected. Then came a discussion on why deep neural networks are attractive for the discovery of complex patterns underlying the large-scale scHi-C data, and how they are evolving with the growing understanding of TAD structures at single-cell level. Furthermore, the challenges that may be encountered at single-cell level were outlined, and an outlook for the emerging trends were delivered in the light of artificial intelligence.

The advancements of machine learning and artificial intelligence methods benefit the biomarker discovery, help to get deep insights into gene regulatory mechanism and genetic disorder, and serve the prognostics, diagnostics, and treatment of diseases. Zhang et al. leveraged transcriptomic analysis and machine learning methods to identify novel biomarkers and investigate the genetic characteristics underlying hypertrophic cardiomyopathy (HCM). The differentially expressed genes (DEGs) were identified and comprehensively analyzed, where the top 12 DEGs were considered as the biomarker ones. And a diagnostic model for HCM was proposed on basis of these biomarker genes by comparing the different combinations and configurations of a total of 12 machine learning algorithms. Yu et al. conducted a dual disease co-expression analysis to reveal the potential roles of estrogen-related genes in postmenopausal osteoporosis (PMO) and Parkinson’s disease (PD). The shared genetic variants between PMO and PD were uncovered with a Bayesian colocalization analysis, and a total of 11 DEGs were identified and went through a bioinformatic pipeline, including KEGG and GO enrichment analysis, PPI network, and TF-gene interaction detection, so that the important signaling pathways and therapeutic targets for PMO and PD were discovered with the help of graph theory, machine learning, and even deep learning methods.

Beyond the spatial organizations of genome and biomarker discovery of diseases, machine learning methods can also be used to optimize DNA barcoding for taxonomic groups, essentially a “fingerprint” for each species depending on one or several short DNA sequences rather than traditional morphological characters. Zhang et al. tried to give out the DNA barcodes for nine species of Syringa trees. The intraspecific and interspecific genetic distances for single and combined DNA sequences were quantified using Kimura two-parameter model, followed by a Wilcoxon signed rank test to score the significance of differences between these species. Besides, the BLAST searches and sequence character analysis were carried out, and a neighbor-joining tree was constructed to cluster these nine species into distinct clades. The results demonstrate that the combination of ITS2 + psbA-trnH + trnL-trnF can be regarded as an optimal barcode for identifying the nine species of Syringa trees. Nevertheless, there is still a lot of room for the development and application of machine learning and even deep learning methods in this field.

Taken together, several studies on bioinformatic analysis of genomic and transcriptomic data are involved in this Research Topic, including a survey of artificial intelligence applications for TADs on scHi-C data, two machine learning approaches for identification of genomic biomarkers and therapeutic targets of diseases, and a DNA barcoding tool for Syringa species. It is expected that the Research Topic may help to expand insights into structure, function, and disorder of genomes, and demonstrates the power of machine learning, especially artificial intelligence methods, in bioinformatics both at present and in the near future.

